# Sustained visual attentional load modulates audiovisual integration in older and younger adults

**DOI:** 10.1177/20416695231157348

**Published:** 2023-02-23

**Authors:** Yanna Ren, Hannan Li, Yan Li, Zhihan Xu, Rui Luo, Hang Ping, Xuan Ni, Jiajia Yang, Weiping Yang

**Affiliations:** Department of Psychology, College of Humanities and Management, 326770 Guizhou University of Traditional Chinese Medicine, Guiyang, China; University Science Park Management Center, 105797 Guiyang University, Guiyang, China; Department of Psychology, College of Humanities and Management, 326770 Guizhou University of Traditional Chinese Medicine, Guiyang, China; Department of Foreign Language, 165069 Ningbo University of Technology, Ningbo, China; Department of Psychology, College of Humanities and Management, 326770 Guizhou University of Traditional Chinese Medicine, Guiyang, China; Applied Brain Science Lab Interdisciplinary Science and Engineering in Health Systems, 12997 Okayama University, Okayama, Japan; Department of Psychology, Faculty of Education, Hubei University, Wuhan, China

**Keywords:** audiovisual integration (AVI), sustained attention, attentional load, older adults, race model

## Abstract

Previous studies have shown that attention influences audiovisual integration (AVI) in multiple stages, but it remains unclear how AVI interacts with attentional load. In addition, while aging has been associated with sensory-functional decline, little is known about how older individuals integrate cross-modal information under attentional load. To investigate these issues twenty older adults and 20 younger adults were recruited to conduct a dual task including a multiple object tracking (MOT) task, which manipulated sustained visual attentional load, and an audiovisual discrimination task, which assesses AVI. The results showed that response times were shorter and hit rate was higher for audiovisual stimuli than for auditory or visual stimuli alone and in younger adults than in older adults. The race model analysis showed that AVI was higher under the load_3 condition (monitoring two targets of the MOT task) than under any other load condition (no-load [NL], one or three targets monitoring). This effect was found regardless of age. However, AVI was lower in older adults than younger adults under NL condition. Moreover, the peak latency was longer, and the time window of AVI was delayed in older adults compared to younger adults under all conditions. These results suggest that slight visual sustained attentional load increased AVI but that heavy visual sustained attentional load decreased AVI, which supports the claim that attention resource was limited, and we further proposed that AVI was positively modulated by attentional resource. Finally, there were substantial impacts of aging on AVI; AVI was delayed in older adults.

In daily life, a human individual is surrounded by stimuli from various sensory modalities, such as visual, auditory, tactile, olfactory, and gustatory stimuli. To improve perception of the outside world, our brain merges cross-modal information either automatically or voluntarily (Tang et al., 2016) through multisensory integration (Stein, 2012; Stein & Meredith, 1993). Previous human and animal studies have confirmed that the response to cross-modal stimuli is faster and more accurate than that to unimodal stimuli (Meredith & Stein, 1986; Stein, 2012; Ren, Xu, Wang, & Yang, 2020). Vision and hearing are important modalities (Ren, Xu, Wang, et al., 2020), and the integration of information from auditory and visual modalities is called audiovisual integration (AVI). Attention refers to the orientation to and concentration on a certain object in terms of cognitive processing and is a common feature accompanying mental processes such as perception, memory, thinking, and imagination (Petersen & Posner, 2012). Several studies from Talsma's team have shown that attention greatly modulates AVI at multiple stages and that AVI is higher under attended conditions than under unattended conditions (Talsma, Doty, & Woldorff, 2007; Talsma, Senkowski, Soto-Faraco, & Woldorff, 2010; Talsma, Senkowski, & Woldorff, 2009; Talsma & Woldorff, 2005).

According to perceptual load theory, the attentional resources of each individual are limited; that is, if one task occupies more attentional resources, less will be available for other tasks (Lavie, 1995; Lavie & Tsal, 1994). Alsius et al. first examined the interaction between attentional load and AVI using a McGurk paradigm to assess AVI. In this paradigm, the perceived syllable changes if pronunciation (auditory stimulus) is incongruent with the articulation (visual stimulus); for example, the pronunciation “ba” paired with the articulation of “ga” results in the perceived syllable “da” (McGurk & Macdonald, 1976). In Alsius et al.'s studies, under low-attentional load conditions, only the McGurk task was presented; however, under high-attentional load conditions, the McGurk paradigm was accompanied by a rapid serial visual presentation (RSVP) task to occupy attentional resources (Alsius et al., 2007, 2014). Both the behavioral and electroencephalogram results showed that AVI was lower under the high-attentional load condition than under the low-attentional load condition. However, Alsius et al. used semantic materials that required high-level processing to assess AVI, and it is difficult to determine whether the attentional load affected AVI or semantic processing. To clarify this, Ren et al. applied a similar dual task, but assessed AVI using meaningless auditory and visual signals (Ren, Li, Wang, & Yang, 2020). In addition, to further investigate how AVI altered with attentional resources, three visual attentional loads were included. Under the low-attentional load condition, only the AVI task was presented; under medium- and high-attentional load conditions the dual tasks included the audiovisual (AV) discrimination task (to assess AVI) and the RSVP task (to vary attentional resources; Ren, Li, et al., 2020). Their results showed that AVI and global functional connectivity were higher under the medium-attentional load condition than under the low- and high-attentional load conditions, which suggests that AVI was increased under a slight visual attentional load but decreased under a heavy load. Wahn and König reported that the neural bases for auditory attention and visual attention are distinct to some degree (Wahn & König, 2017), especially during stimulus attribute discrimination (Alais, Morrone, & Burr, 2006; Arrighi, Lunardi, & Burr, 2011). To further clarify how auditory attentional load influences AVI, Ren et al. (2021) performed a similar study using a rapid serial auditory presentation (RSAP) task instead of the RSVP task (Ren, Zhao, et al., 2021). They found that similar to that under the visual attentional load condition, AVI increased under slight auditory attentional load but decreased with an increase in attentional load.

In the studies conducted by Alsius et al. ( Alsius et al., 2005, 2014) and Ren et al. (Ren, Li, et al., 2020; Ren, Zhao, et al., 2021), transient attention was elicited, due to frequent switching of attention to brief, fleeting tasks or stimuli (the RSAP or RSVP tasks). In contrast, sustained attention requires focused attention to a single stimulus (Smid, de Witte, Homminga, & van den Bosch, 2006). A previous study showed that transient and sustained attention involved different top-down and bottom-up attention; top-down control was engaged in sustained attention tasks, but both top-down and bottom-up control were engaged for transient attention tasks (Di Russo et al., 2021). To fully determine the interaction between attention and AVI, it is necessary to explore how AVI changes in response to differences in sustained attentional load. Talsma et al.'s studies have confirmed higher AVI in the attended condition than in the unattended condition (Talsma et al., 2007,  2009, 2010; Talsma & Woldorff, 2005), and tasks of appropriate levels of difficulty can produce optimal performance (Kamijo, Nishihira, Higashiura, & Kuroiwa, 2007; Yerkes & Dodson, 1908). Therefore, we hypothesized that AVI is higher under a high sustained attentional load than under a low sustained attentional load. In addition, according to perceptual load theory (Lavie, 1995; Lavie & Tsal, 1994), during dual tasks, if one task captures more attentional resources, less will be available for the other task. Therefore, we further hypothesized that AVI is decreased under excessive attentional load. In the current study, a dual-task paradigm was applied in which AVI was assessed using an AV discrimination task, and sustained visual attention was controlled using a multiple object tracking (MOT) task (Wahn & König, 2017). We generated four attentional load conditions and tested our hypotheses by comparing AVI under different attentional load conditions. Specifically, according to our hypotheses, AVI would be higher under the medium-attentional load condition than under the low- and high-attentional load conditions.

In addition to the effect of attention, we also investigated the effects of aging on AVI. AVI in older adults was altered compared with younger adults, which was attributed to the functional decline in auditory and visual processing with aging (Ren, Xu, Wang, et al., 2020; Stein, 2012). In contrast, some studies have reported that as a compensatory mechanism, AVI is higher in older adults than in younger adults (Diederich, Colonius, & Schomburg, 2008; Laurienti, Burdette, Maldjian, & Wallace, 2006; Peiffer, Mozolic, Hugenschmidt, & Laurienti, 2007). A contrary conclusion was obtained by other studies (Mahoney, Li, Oh-Park, Verghese, & Holtzer, 2011; Stephen, Knoefel, Adair, Hart, & Aine, 2010; Tye-Murray, Sommers, Spehar, Myerson, & Hale, 2010); this discrepancy mainly resulted from differences in experimental materials, study paradigms, and analysis methods (Ren, Xu, Lu, Wang, & Yang, 2020; Ren, Xu, Wang, et al., 2020; Yang, Li, Li, Guo, & Ren, 2020). Using the same experimental materials, study paradigms, and analysis methods, Ren et al. (2020, 2021) investigated the difference in AVI between older and younger adults (Ren, Hou, et al., 2021; Ren, Li, et al., 2020) and the difference in AVI under visual (Ren, Li, et al., 2020) and auditory (Ren, Li, et al., 2020) transient attentional load conditions. Their results showed that AVI was lower and delayed in older adults compared to younger adults when integrating peripheral stimuli. In addition, similar to younger adults, under the visual transient attentional load condition, AVI was higher under the medium-load condition than under the low- and high-load conditions, and AVI was decreased with increases in transient auditory attentional load. However, differences in the AVI of older adults under sustained attentional load are unclear; therefore, another aim of the current study was to determine the effect of aging on AVI by comparing the AVI of older and younger adults under all attentional load conditions. Considering age-related attentional declines in older adults (Fraser & Bherer, 2013; Williams et al., 2016) and higher AVI under the attended condition than under the unattended condition (Talsma et al., 2007, 2009,  2010; Talsma & Woldorff, 2005), we hypothesized that AVI is reduced in older adults.

## Methods

### Subjects

Twenty older adults (55–69 years; mean age ± standard deviation [*SD*], 60.2 ± 4.0) and 20 younger adults (18–22 years; mean age ± *SD*, 19.2 ± 1.0) were recruited to participate in the current study. The older adults were recruited from Guiyang City, and the younger adults were college students at Guizhou University of Traditional Chinese Medicine. All participants were free of neurological diseases, had normal hearing, had normal or corrected-to-normal vision, were right-handed, and were naive to the purpose of the experiment. The Mini-Mental State Examination scores of the older (scores: 26–30) and younger (scores: 29–30) adults fell within 2.5 *SD* for their mean age and education level (Bravo & Hébert, 1997). All participants reported no history of cognitive disorders and successfully completed the experiment. Before participating in the experiment, all participants provided written informed consent; all experimental procedures were approved by the Second Affiliated Hospital of Guizhou University of Traditional Chinese Medicine.

### Stimuli

**Figure 1.**
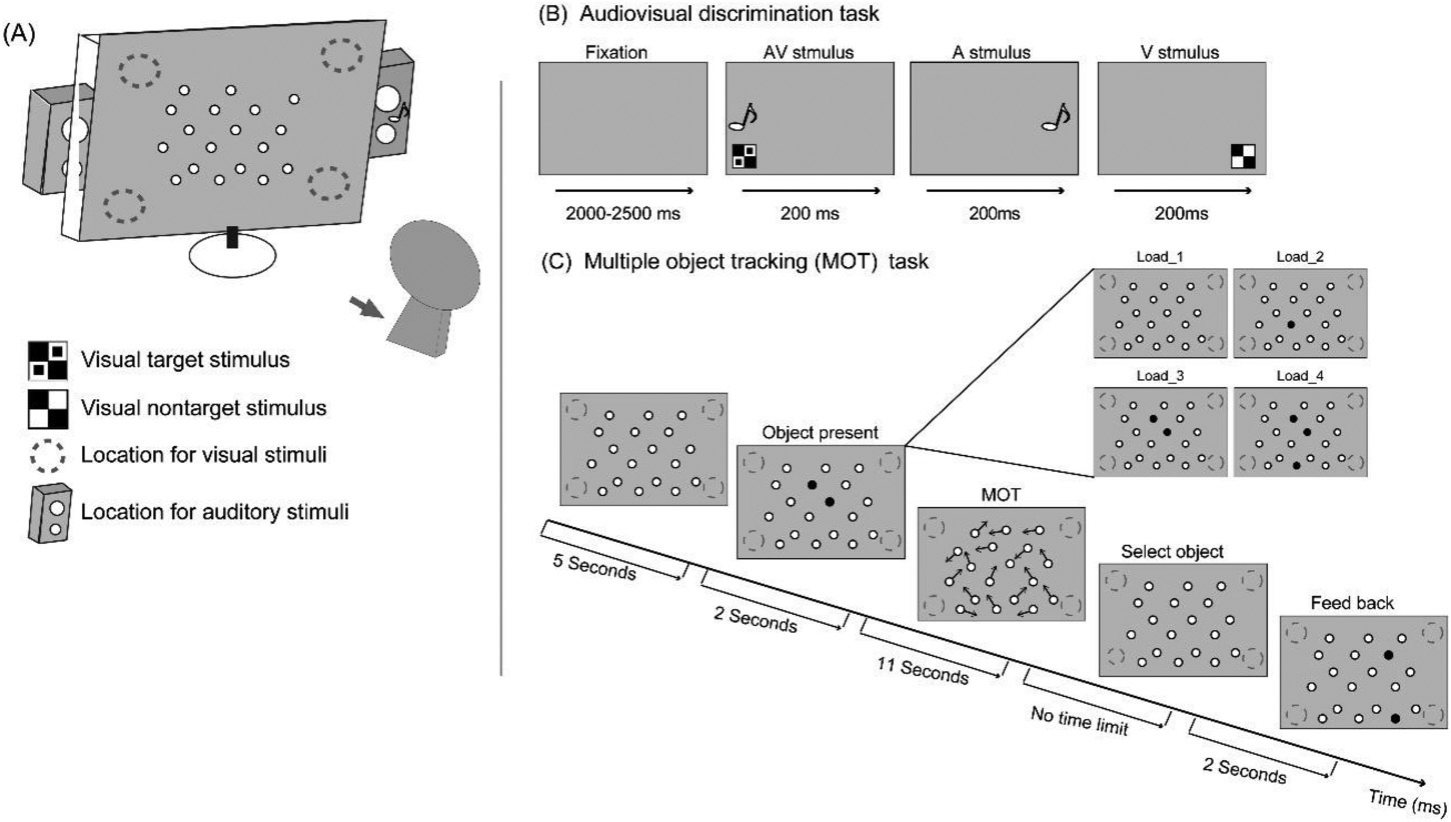
The experimental scenario (A); a representative sequence from the audiovisual discrimination task (B) and a representative sequence for the multiple object tracking (MOT) task in the load_3 condition (other attentional load conditions are also indicated) (C). The AV discrimination task and MOT task were presented simultaneously or independently according to the attentional load condition.

Similar to our previous study on auditory attentional load (Ren, Zhao, et al., 2021), a dual task was employed in the current study including the following two tasks: an AV discrimination task for evaluating AVI (Ren, Li, et al., 2020) and an MOT task for manipulating sustained visual attentional load (Wahn & König, 2017; Figure 1A). The two tasks were presented simultaneously or independently according to the attentional load condition.

For the AV discrimination task, the auditory nontarget was a 1,000-Hz 60-dB sinusoidal tone, and the auditory target was 60-dB white noise. The visual nontarget was a black and white checkerboard image (B/W checkerboard image, 52 × 52 mm, with a visual angle of 5°), and the visual target was a B/W checkerboard image with a square black dot within each white square of the checkerboard (Figure 1B). The AV nontarget was a simultaneous presentation of the visual nontarget and auditory nontarget, and the AV target was the simultaneous presentation of the visual target and auditory target. There were no other combinations of auditory and visual stimuli. The visual stimuli (V) were presented on a computer monitor at a 60-cm distance from participants’ eyes in the upper/lower left or right quadrant of the screen for 200 ms with a 12-degree visual angle (Figure 1B). Auditory stimuli (A) were presented through two speakers located on the left and right sides of the monitor at approximately 60 dB Sound Pressure Level for a duration of 200 ms (10 ms of the rise/fall cosine gate). For the MOT task (Figure 1C), as in a previous study (Wahn & König, 2017), 18 small balls with a visual angle of 1° were included.

### Procedure

The stimulus presentations and data collection were controlled using PsychoPy. To fully understand how attentional load influences AVI, AVI was assessed in five different load conditions. In all load conditions, the AV discrimination task was identical (Figure 1B), but the presentation of the MOT task and the reactive mode were purposively controlled. In the no-load (NL) condition, only the AV discrimination task was presented; however, the MOT task was presented simultaneously with the AV discrimination task in the other attentional load conditions.

For the AV discrimination task, the A, V, and AV stimuli were randomly presented with a random interstimulus interval of 2,000 to 2,500 ms (Figure 1B). The participants were instructed to press the left button of the mouse to respond to the target stimuli as rapidly and as accurately as possible. In total, there were 60 trials for each target stimulus type (A, V, and AV) and 20 trials for each nontarget stimulus type (A, V, and AV) in each attentional load session (240 trials).

For the MOT task, 18 small white balls were presented on the screen in random locations for 5 s, and then one (load_2 condition), two (load_3 condition), or three (load_4 condition) of them turned black for 2 s (Figure 1C). Subsequently, the black ball(s) turned white, and all balls moved in a disorderly fashion for 11 s and then stopped. The participant was instructed to identify which of the white balls had previously turned black. Following the participant's identification, feedback was presented.

In the NL condition, the MOT task was not presented, and the participant was only instructed to respond to the targets of the AV discrimination task. In the attentional load_1 condition, the stimuli of the AV discrimination task were presented randomly during the phase in which the small balls moved in a disorderly fashion (11 s). For each trial of the MOT task, four random stimuli from the AV discrimination task were presented. The participants were instructed to respond only to the target in the AV discrimination task but to withhold responses associated with the MOT task. In the load_2 condition, for the MOT task, one small ball turned black, and the AV discrimination task was the same as that in the load_1 condition. The participant was instructed to respond to the target of the AV discrimination task while monitoring the MOT task and then to identify the target in the MOT task. In the load_3 and load_4 conditions, the presentation of the dual task was similar to that in the load_2 condition, but two small balls (in the load_3 condition) or three small balls (in the load_4 condition) turned black.

### Analysis

The hit rates and response times (RTs) were computed separately for each subject under each condition (Table 1), and then, the data were submitted to a 2 (group: older, younger) × 5 (attentional load: NL, load_1, load_2, load_3, and load_4) × 3 (stimulus: A, V, and AV) analysis of variance (ANOVA). The statistical significance level was set at *p* ≤ .05, and the effect size estimates, *η_p_^2^,* are also reported (Greenhouse‒Geisser corrections with corrected degrees of freedom).

**Table 1.** Mean response time (ms) and hit rate (%) for older and younger adults in each attentional load condition.

	Older adults								Younger adults						
	Response time				Hit rate				Response time				Hit rate		
	V	A	AV		V	A	AV		V	A	AV		V	A	AV
No-load	549 (44)	609 (49)	508 (47)		90.8 (4.4)	91.6 (4.1)	90.0 (3.7)		575 (44)	515 (71)	422 (51)		96.0 (3.8)	98.5 (1.7)	99.8 (0.8)
Load_1	579 (36)	581 (59)	504 (44)		87.3 (7.6)	94.4 (3.3)	93.7 (3.3)		503 (46)	502 (62)	430 (46)		97.3 (2.0)	98.7 (2.0)	99.9 (0.5)
Load_2	635 (51)	639 (68)	556 (60)		87.4 (5.2)	92.9 (4.4)	89.9 (3.6)		498 (42)	491 (65)	423 (46)		98.5 (1.5)	99.0 (2.0)	99.9 (0.1)
Load_3	690 (66)	691 (84)	581 (57)		86.8 (7.0)	90.9 (5.4)	91.6 (3.7)		511 (49)	506 (67)	438 (55)		98.8 (1.7)	99.1 (2.8)	99.8 (0.8)
Load_4	712 (78)	669 (65)	592 (52)		82.9 (6.4)	85.1 (6.0)	91.8 (4.0)		537 (71)	506 (78)	442 (61)		96.2 (5.2)	98.3 (2.4)	99.5 (1.2)

Data are presented as mean (standard deviation).

As in our previous study of auditory attentional load (Ren, Zhao, et al., 2021), the occurrence of AVI was assessed using a race model by cumulative distribution functions (CDFs; Miller, 1982, 1986). The race model (P_RM_) is a statistical prediction model, P_RM _= (P_A _+ P_V_) − P_A _× P_V_, based on the CDFs of the unimodal visual condition (P_V_) and unimodal auditory condition (P_A_), allowing a direct comparison with the bimodal AV condition (P_AV_). P_A_, P_V_, and P_AV_ are the probability of responding within a given time in a unimodal visual trial, unimodal auditory trial, and bimodal AV trial, respectively. If P_RM_ was significantly different from P_AV_, AVI is considered to occur. To assess the amount of AVI in various conditions, a difference probability curve was generated by subtracting a subject's race model CDF from his or her AV CDF in each 10-ms bin (Hugenschmidt, Mozolic, & Laurienti, 2009; Laurienti et al., 2006; Laurienti, Kraft, Maldjian, Burdette, & Wallace, 2004; Peiffer et al., 2007). The peak of the difference probability curve (peak benefit) was computed separately for each participant in each condition to assess the amount of AVI. The time point of peak benefit was defined as the peak latency and the time interval at which a significant difference between the AV CDF and the race model CDFs occurred was defined as the AVI time window, which was used to assess when AVI occurred.

## Results

### Hit Rates and RTs

A 2 (group: older, younger) × 5 (attentional load: NL, load_1, load_2, load_3, and load_4) × 3 (stimulus type: A, V, and AV) ANOVA for hit rates revealed significant main effects of the group, *F*(1, 38) = 314.961, *p *< .001, *η_p_^2^*= 0.892, attentional load, *F*(4, 152) = 10.545, *p *< .001, *η_p_^2^*= 0.217, and stimulus type, *F*(2, 76) = 31.249, *p *< .001, *η_p_^2^*= 0.451; specifically, the hit rate was lower for older adults than for younger adults for A and V stimuli than for AV stimuli under the load_4 condition than under other attentional load conditions. The interactions between group and stimulus type, *F*(2, 76) = 4.688, *p *= .021, *η_p_^2^*= 0.110, and between group and attentional load, *F*(4, 152) = 8.120, *p *< .001, *η_p_^2^*= 0.176, were significant. Post hoc analysis showed that the hit rate was lower for older adults than for younger adults under all attentional load and stimulus types (all *ps *< .001). For younger adults, the hit rate for V stimuli was significantly lower than for AV stimuli (*p* = .006) but not for A stimuli (*p* = .067), and there was no significant difference between attentional load conditions. However, for older adults, the hit rate for V stimuli was significantly lower than that for AV (*p *< .001) and A (*p *< .001) stimuli and lower under the load_4 condition than under the other conditions (all *p*s* *≤ .004). In addition, there were significant interactions of attentional load × stimulus type, *F*(8, 304) = 4.550, *p *< .001, *η_p_^2^*= 0.107, and group × attentional load × stimulus type, *F*(8, 304) = 5.172, *p *< .001, *η_p_^2^*= 0.120. Post hoc analysis showed that for younger adults, no significant difference was found between attentional load condition and stimulus type. For older adults, the lowest hit rate was found when responding to the V stimulus under the load_1 and load_3 conditions (AV = A > V), but in response to the A stimulus under the load_2 and load_4 conditions (AV = V > A). In addition, for older adults, no significant difference was found among load conditions when responding to AV stimuli, but there was a significantly lower hit rate for A and V stimuli under the load_4 condition than other conditions (all *p*s* *≤ .013).

A 2 (group: older, younger) × 5 (attentional load: NL, load_1, load_2, load_3, and load_4) × 3 (stimulus type: A, V, and AV) ANOVA for RTs revealed significant main effects of the group, *F*(1, 38) = 85.107, *p *< .001, *η_p_^2^*= 0.691, attentional load, *F*(4, 152) = 36.029, *p *< .001, *η_p_^2^*= 0.487, and stimulus type, *F*(2, 76) = 123.631, *p *< .001, *η_p_^2^*= 0.765; specifically that the response was slower for older than for younger adults for A and V stimuli than for AV stimuli under load_3 and load_4 conditions than under the other conditions. The interaction between group and attentional load was significant, *F*(4, 152) = 19.819, *p *< .001, *η_p_^2^*= 0.343. Post hoc analysis showed that the response of older adults was slower than that of younger adults under all attentional load conditions (all *p*s < .001). For younger adults, no significant difference was found among the attentional load conditions (all *p*s ≥ .262); however, the response was slower under load_3 and load_4 conditions than under the other conditions (NL = load_1 > load_2 > load_3 = load_4). In addition, the interaction between attentional load and stimulus type was also significant, *F*(8, 304) = 18.995, *p *< .001, *η_p_*^2^= 0.333. Post hoc analysis showed that the response to the A stimulus was slower than to the V stimulus under the NL condition (AV > A > V, all *p*s < .001), compared to the response to the V stimulus under the load_1, load_2, and load_3 conditions (AV > A = V), but faster than the response to the V stimulus under the load_4 condition (AV > V > A, all *p*s ≤ .006). In addition, the response to the V stimulus was faster than that to the A stimulus under the NL condition and load_1 condition (AV > V > A, all *p*s ≤ .009) but slower under the load_2, load_3, and load_4 conditions (AV > A > V, all *p*s ≤.028).

### Race Model

**Figure 2.**
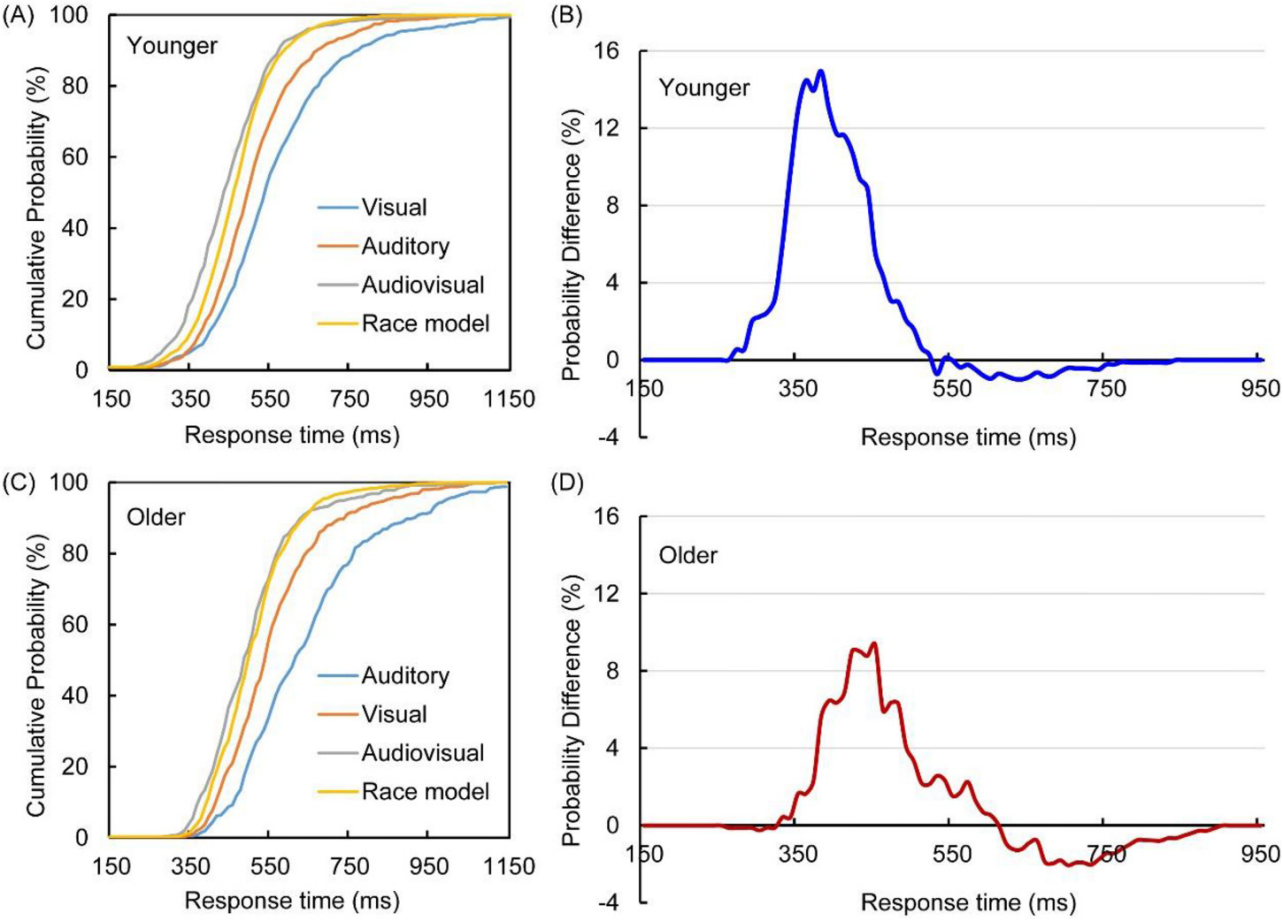
Cumulative distribution functions (CDFs) for the discrimination response times to auditory, visual, and audiovisual stimuli and the race model in younger (A) and older (C) adults under the no-load condition. Probability difference between audiovisual CDFs and race model CDFs in younger (B) and older (D) adults under the no-load condition.

The race model was calculated using the CDFs of A and V stimuli (Figure 2A and C), and AVI was assessed using the probability difference generated by subtracting race model CDFs from AV CDFs, as shown in Figure 2B for younger adults and Figure 2D for older adults under the NL condition.

**Figure 3.**
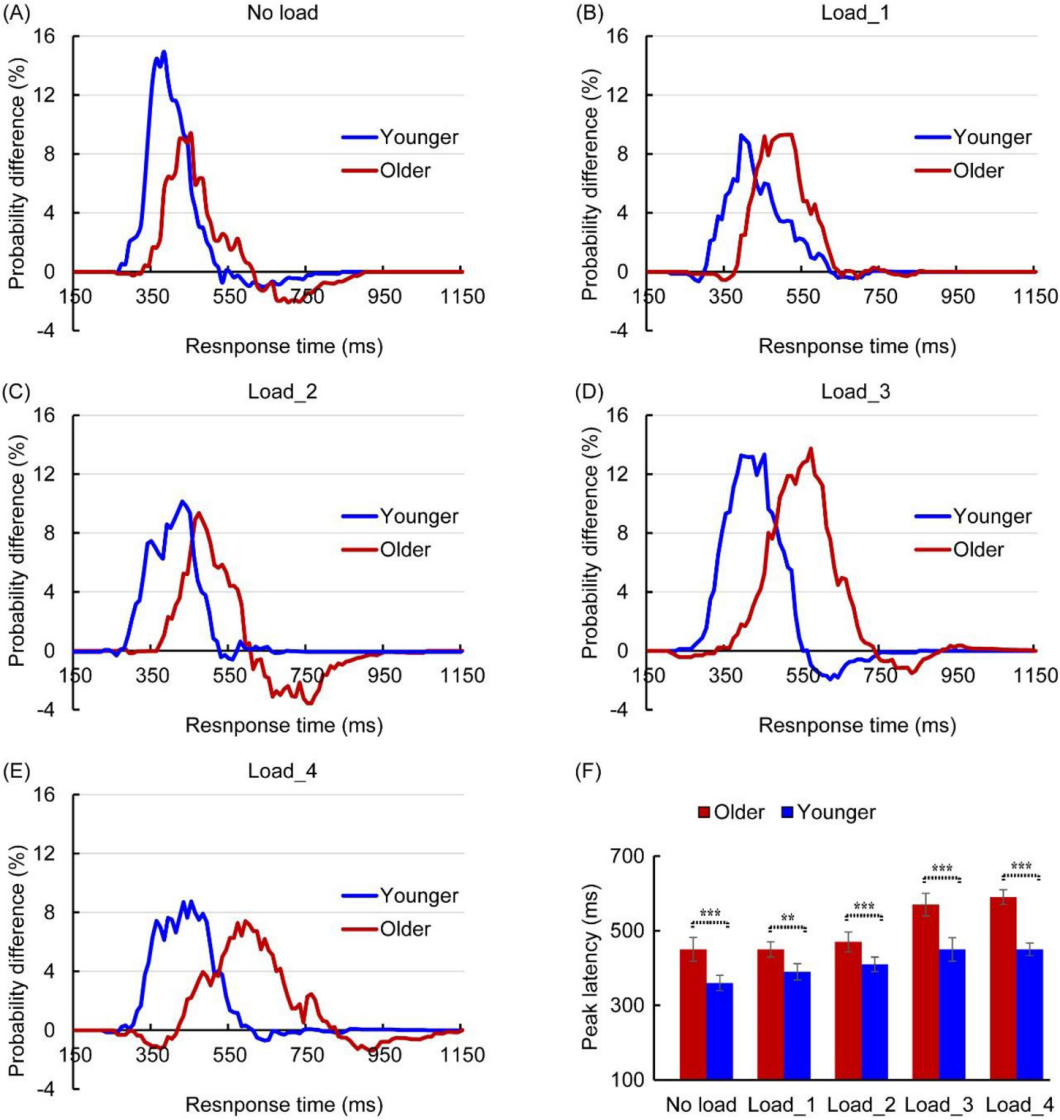
Comparison of probability differences between younger and older adults in the no-load (NL) (A), load_1 (B), load_2 (C), load_3 (D), and load_4 (E) conditions. The peak latency was longer for older adults than for younger adults (F). **p < .01, ***p < .001.

Significant AVI was found under all attentional load conditions for both younger and older adults (Figure 3). The 2 (group: older, younger) × 5 (attentional load: NL, load_1, load_2, load_3, and load_4) ANOVA for peak benefit showed a significant main effect of attentional load, *F*(4, 152) = 4.273, *p *= .004, *η_p_^2^*= 0.101, with AVI higher under the NL and load_3 conditions than under the load_1, load_2, and load_4 conditions (all *p*s ≤ 0.027). There was a significant interaction between group and attentional load, *F*(4, 152) = 7.270, *p *= .032, *η_p_*^2^ = 0.287. Post hoc analysis revealed that AVI was higher for younger adults than for older adults under the NL condition (14.9% vs. 9.4%, *p* < .001) but comparable under the load_1 (9.3% vs. 9.3%, *p* = .612), load_2 (10.2% vs. 9.4%, *p *= .053), load_3 (13.3% vs. 13.7%, *p* = .091), and load_4 conditions (8.4% vs. 7.4%, *p *= .083; Table 2). In addition, for younger adults, AVI was higher under the NL and load_3 conditions (NL = load_3 > load_2 > load_1 = load_4) than under the other conditions, but no significant difference was found between the NL and load_3 conditions (*p* = .142). However, for older adults, AVI was higher under load_3 conditions (load_3 > NL = load_1 = load_2 > load_4) than under the other conditions. There was no significant main effect of the group, *F*(1, 38) = 1.794, *p *= .188, *η_p_^2^*= 0.045.

**Table 2.** The peak benefit (%), peak latency (ms), and time window (ms) of audiovisual integration for older and younger adults under each attentional load condition.

	Peak benefit (%)			Peak latency (ms)			Time window (ms)	
	Older	Younger		Older	Younger		Older	Younger
No-load	9.4	14.9		450	360		390–570	290–490
Load_1	9.3	9.3		450	390		370–590	340–510
Load_2	9.4	10.2		470	410		390–530	310–490
Load_3	13.7	13.3		570	450		410–650	300–520
Load_4	7.4	8.7		590	450		460–600	340–490

A 2 (group: older, younger) × 5 (attentional load: NL, load_1, load_2, load_3, and load_4) ANOVA for peak latency showed a significant main effect of group, *F*(1, 38) = 17.345, *p *< .001, *η_p_*^2^ = 0.313, with a longer peak latency in older adults than in younger adults under all attentional load conditions (all *p*s ≤ .014, Figure 3F). However, there was no significant main effect of attentional load, *F*(4, 152) = 0.969, *p *= .335, *η_p_*^2^ = 0.025, or interaction between group and attentional load, *F*(4, 152) = 0.728, *p *= .405, *η_p_^2^*= 0.019.

In addition, a pairwise comparison between race model CDF and AV CDF in each 10-ms bin was performed, and time intervals with a significant difference were defined as the time windows of AVI. As shown in Table 2, the AVI time window was delayed for older adults compared with younger adults, suggesting a delayed AVI for older adults. In addition, the time window was delayed under the attentional load conditions compared with the NL condition, suggesting that attentional load also delayed AVI.

## Discussion

To clarify how sustained auditory attention and aging modulate AVI, a dual task was applied in which attentional resources were manipulated by the MOT task and AVI was assessed by the AV discrimination task. AVI was higher under the load_3 condition than under the load_1, load_2, and load_4 conditions for both younger and older adults. In addition, AVI was lower in older adults than in younger adults under the NL condition but comparable under the other attentional load conditions. Additionally, the peak latency was longer, and the time window of AVI was delayed in older adults compared to younger adults under all conditions. Moreover, AVI was delayed under all attentional load conditions compared to that under the NL condition.

Consistent with our previous assumption, AVI was higher under the medium-attentional load condition (load_3) than under the low- (load_1 and load_2) and high- (load_4) visual sustained attentional load conditions for both younger and older adults. In the present study, the participant was instructed to perform an MOT task while AVI was simultaneously assessed; this dual task occupied attentional resources to varying extents. In the load_1 condition, although the MOT task was presented, the participant was instructed to respond only to the AV discrimination task; however, in the remaining attentional load conditions, participants were instructed to simultaneously track one, two, and three target balls (in the load_2, load_3, and load_4 conditions, respectively). Studies have found that appropriate difficulty can produce optimal performance, which is consistent with physiological arousal showing an inverted “U”-shaped curve (Kamijo et al., 2007; Yerkes & Dodson, 1908). In the low-attentional load condition (load_1 and load_2), physiological arousal is relatively low, as are the attentional demands of the experimental task. However, as task difficulty increased, more attentional resources were recruited in the medium-attentional load condition (load_3) (Petersen & Posner, 2012). Previously, the AVI was observed to be higher in the attended condition than in the unattended condition (Talsma et al., 2007, 2009, 2010; Talsma & Woldorff, 2005); therefore, it is reasonable that in the present study, AVI was higher under the load_3 condition than under the load_1 and load_2 conditions. According to the perceptual load theory of Lavie et al., the attentional resources of individuals are limited (Lavie, 1995; Lavie & Tsal, 1994); as the MOT task difficulty increased (load_4), less or insufficient attentional resources were available for the AV discrimination task, which further led to lower AVI under the load_4 condition than under the load_3 condition.

Additionally, AVI was obviously delayed in older adults compared to younger adults, which is consistent with numerous previous studies (Laurienti et al., 2006; Ren, Li, et al., 2020; Ren, Xu, Lu, et al., 2020). It is well known that there is a general functional decline with aging (Anderson, 2019; Schupf et al., 2005), which further leads to a slower response to many cognitive tasks (Anderson, 2019; Freiherr, Lundström, Habel, & Reetz, 2013). Colonius and Diederich proposed that AVI occurs when the processing of auditory and visual information is completed within a given time course (Colonius & Diederich, 2004; Diederich et al., 2008). Therefore, the functional decline in auditory and visual information processing might be the most likely reason for delayed AVI in older adults.

Consistent with our previous studies (Mahoney et al., 2011; Ren, Hou, et al., 2021; Stephen et al., 2010; Tye-Murray et al., 2010), AVI was lower in older adults than in younger adults under the NL condition. However, other studies have reported an enhanced AVI in older adults (Diederich et al., 2008; Laurienti et al., 2006; Peiffer et al., 2007). Compared with younger adults, studies have found a significant attentional decline in older adults, who have a decreased ability to suppress interfering stimuli (Fraser & Bherer, 2013; Williams et al., 2016). As AVI was found to be higher in the attended condition than in the unattended condition (Talsma et al., 2010; Tang et al., 2016), the age-related attentional decline might be the main factor leading to the reduced AVI in older adults. In addition, in studies that found enhanced AVI in older adults, the visual stimuli were presented centrally (Diederich et al., 2008; Laurienti et al., 2006; Peiffer et al., 2007); however, in the current study, the visual stimuli were presented with a 12° visual angle (horizontal and vertical). With aging, the information processing ability for peripheral information is decreased (Wang et al., 2017); therefore, another possible reason for the reduced AVI in older adults might be the presentation location of visual stimuli.

However, inconsistent with our hypothesis, under attentional load conditions (load_1, load_2, load_3, load_4), the AVI of older adults was comparable to that of younger adults. Ren et al. (2021) investigated how auditory attentional load affects AVI (Ren, Hou, et al., 2021) and found that the AVI of older adults was lower than that of younger adults under all attentional load conditions. Studies have found that visual and auditory attentional resources during stimulus attribute discrimination are distinct (Wahn & König, 2017) and that auditory distractors attract attention more easily than visual distractors (Ren, Zhang, et al., 2021). In the study by Ren et al. (2021), an auditory attentional load was applied, but in the current study, a visual attentional load was applied; visual dominance during AVI has been extensively reported (Diaconescu et al., 2013). Therefore, the conflicting results might be mainly attributed to differences in the neural mechanisms of visual and auditory attentional resources; these mechanisms need to be further investigated. Additionally, studies have found that although AVI in older adults was lower behaviorally, their global functional connectivity was higher than that in younger adults during AV information processing (Ren, Guo, et al., 2020; Wang et al., 2017, 2018). Diaconescu, Hasher, and McIntosh, (2013) and Ren, Li, et al. (2020) found that older adults recruited additional brain regions during the processing of AV stimuli. Considering the lower AVI in older adults compared to younger adults under the NL condition and the attentional deficit with aging (Fraser & Bherer, 2013; Williams et al., 2016), the compensatory mechanism (Anderson, 2019; Ren, Xu, Wang, et al., 2020) during AVI under a visual attentional load in older adults might be the main factor contributing to their comparable AVI to that in younger adults, but further neuroimaging studies are needed.

In conclusion, the moderate attentional load increased AVI due to optimal levels of physiological arousal, but AVI was reduced under higher attentional load conditions, which was attributed to the lack of available attentional resources. Additionally, AVI was delayed in older adults compared to younger adults, and attentional load also delayed AVI.
